# Geographical distribution and driving force of micro-eukaryotes in the seamount sediments along the island arc of the Yap and Mariana trenches

**DOI:** 10.1128/spectrum.02069-23

**Published:** 2023-11-09

**Authors:** Yue Zhang, Hongbin Liu, Ning Huang, Xiaotong Peng, Hongmei Jing

**Affiliations:** 1 CAS Key Lab for Experimental Study under Deep-sea Extreme Conditions, Institute of Deep-sea Science and Engineering, Chinese Academy of Sciences, Sanya, China; 2 Department of Ocean Science, The Hong Kong University of Science and Technology, Clear Water Bay, Kowloon, Hong Kong, China; 3 Southern Marine Science and Engineering Guangdong Laboratory, Zhuhai, China; 4 HKUST-CAS Sanya Joint Laboratory of Marine Science Research, Chinese Academy of Sciences, Sanya, China; Chinese Academy of Sciences, Beijing, China

**Keywords:** micro-eukaryote, driving force, trophic state, seamount

## Abstract

The specific topographic characteristics and complex hydrodynamics of seamounts could directly or indirectly affect the distribution and trophic status of microbes. However, little is known about the distribution patterns and associated driving forces of micro-eukaryotes in the deep seamounts. Micro-eukaryotes in the seamount sediments along the island arc of the Yap and Mariana trenches were investigated using high-throughput sequencing and quantitative PCR based on the 18S rRNA gene. Micro-eukaryotic communities from seamounts were clustered together and distinct from those of the depression, which showed comparatively lower diversity, gene abundance, endemic species, and higher proportions of decomposers and parasites. This clear geographical distribution pattern was mainly shaped by the deterministic process, especially environmental (61.63%) and biotic (mainly the unexplained 30.05%) factors, potentially reflecting the seamount effect along the horizontal dimension very likely caused by enclosed circulation cells. Varied community connectivity existed along the summit, flank, and base of different seamounts, and this seamount effect along the vertical dimension might be attributed to the upwelling/downwelling water flows. Prevalence of parasitism and predation for the trophic relationships among micro-eukaryotes would be helpful for adaptation, diversification, and maintaining the ecological balance in this extreme biosphere. This study provides insights into the ecological patterns, assembling processes, and species interactions underlying the dynamics of micro-eukaryotic communities across a series of seamounts with a comparison of the depression and would expand our understanding of seamount effects on the deep-sea ecosystems.

**IMPORTANCE:**

A distinct distribution pattern was shaped by a deterministic process. Enhanced vertical connectivity expanded the previous understanding of seamount effects. Parasitism and predation were prevalent in the seamounts.

## INTRODUCTION

Micro-eukaryotes, a microbial group of eukaryotes with a body size range of 20–180 μm (1), play key roles in marine ecosystems, functioning as primary producers, consumers, decomposers, and parasites (2). Their community composition and distribution were influenced by different driving forces, such as deterministic and stochastic processes (3). Deterministic processes, comprising variable selection and homogeneous selection, result from the abiotic environmental factors, spatially related processes, and biotic interactions between individuals. Stochastic processes due to random changes (birth, death, immigration and emigration, spatiotemporal variation, and/or historical contingency) in community structure consist of dispersal limitation, homogeneous dispersal, and drift (4). By far, the geographical distribution patterns of micro-eukaryotes have long been debated (5). Micro-eukaryotes had been thought to be widespread or even cosmopolitan, due to their high abundance (6) and lack of clear geographic clustering for marine benthic ciliates and flagellates on a global scale (7, 8). On the other hand, majority (>90%) of micro-eukaryotes proposed with moderate and low abundances were biogeographically restricted (9). Geographically distinct micro-eukaryotic communities have been revealed from the deep-sea sediments (10) and seawaters (11) in different oceanic regions. Biogeography (clustering and separation) and connectivity were the fundamental questions in the field of microbial ecology and could deepen our understanding on the formation of global biodiversity and the underlying shaping processes (12). Therefore, studies on biogeographic distribution and connectivity of micro-eukaryotes are important and necessary.

Seamounts are formed at plate boundaries by the volcanic and tectonic activities of mid-ocean ridges and transform faults (13). They are generally defined as undersea topographic structures with over 1,000 m in height, usually associated with enhanced vertical mixing, Taylor columns, and mesoscale ocean eddies (14). The specific topographic characteristics and complex hydrodynamics of seamounts directly or indirectly enrich the concentrations of particle organic matter and inorganic nutrients and subsequently promote the metabolic activities of microbes (15). Therefore, seamounts generally harbor more microbial species and higher biomass than surrounding waters (16), establishing biological hotspots in the ocean (17), known as “seamount effects.” By far, most studies on seamount were limited to prokaryotes, zooplankton, and fish (18 – 20), while knowledge on micro-eukaryotes was very few (21, 22). In addition, different seamounts are often hydrographically distinct to support unique microbial communities (23). By far, the distribution patterns, connectivity, and driving force of micro-eukaryotic communities among different seamounts were still largely unknown.

The highly diverse trophic status of micro-eukaryotes helped them to fulfill various roles in different marine microbial ecosystems (24). Micro-eukaryotes act as potential intracellular symbionts, and parasites have been revealed in deep-sea cold seeps (25), hydrothermal vents (26), and anoxic fjord (27). These special trophic statuses could enhance the connectivity between species and make carbon transfers more efficient, representing a potentially adaptive strategy to the deep-sea extreme biosphere (28). Seamount is a unique deep-sea niche, where the trophic roles of micro-eukaryotes and their response to the seamount effects remain largely unexplored, although parasitic/symbiotic/endophytic fungi have been detected (21).

The Yap and Mariana trenches, formed by the collision of plates, are both located in the western Pacific Ocean, and the southern end of the Mariana Trench is intersected by the north-south trending Yap Trench (29). Yap-Mariana Junction cuts across the Mariana Ridge and Yap Ridge and is located just to the west of the Mariana Trench. A series of seamounts are located on the island arc of the Yap and Mariana trenches and formed by volcanic magmatic activity associated with plate subduction and compression (23). By far, study on the microbial ecology of the Yap-Mariana seamount has been mainly focused on prokaryotes (20, 30), and the only micro-eukaryotic study has been conducted in the Magellan Seamount in the western Pacific Ocean (22), while micro-eukaryotes across multiple seamount habitats have never been investigated. To bridge the knowledge gap, a series of seamounts along the island arc of the Yap and Marian Trenches were selected, including sediment samples from the summit, to the flank, and to base along the vertical scale, to investigate the spatial variation of micro-eukaryotes based on high-throughput DNA sequencing with a comparison of sediment samples from the depression, which includes a southwest Mariana rift and the Challenger Deep located in the island arc of the Marina Trench. We aimed to reveal (i) the geographical variation of the micro-eukaryotic community in different regions along the island arc, (ii) the connectivity and potential trophic status of micro-eukaryotes along the inter-seamounts and vertical scales, and (iii) the relative contribution of different driving forces, i.e., environmental factors, spatial variables, and bio-interactions, to the formation of micro-eukaryotic communities.

## RESULTS

### Hydrographic conditions

**Fig 1:**
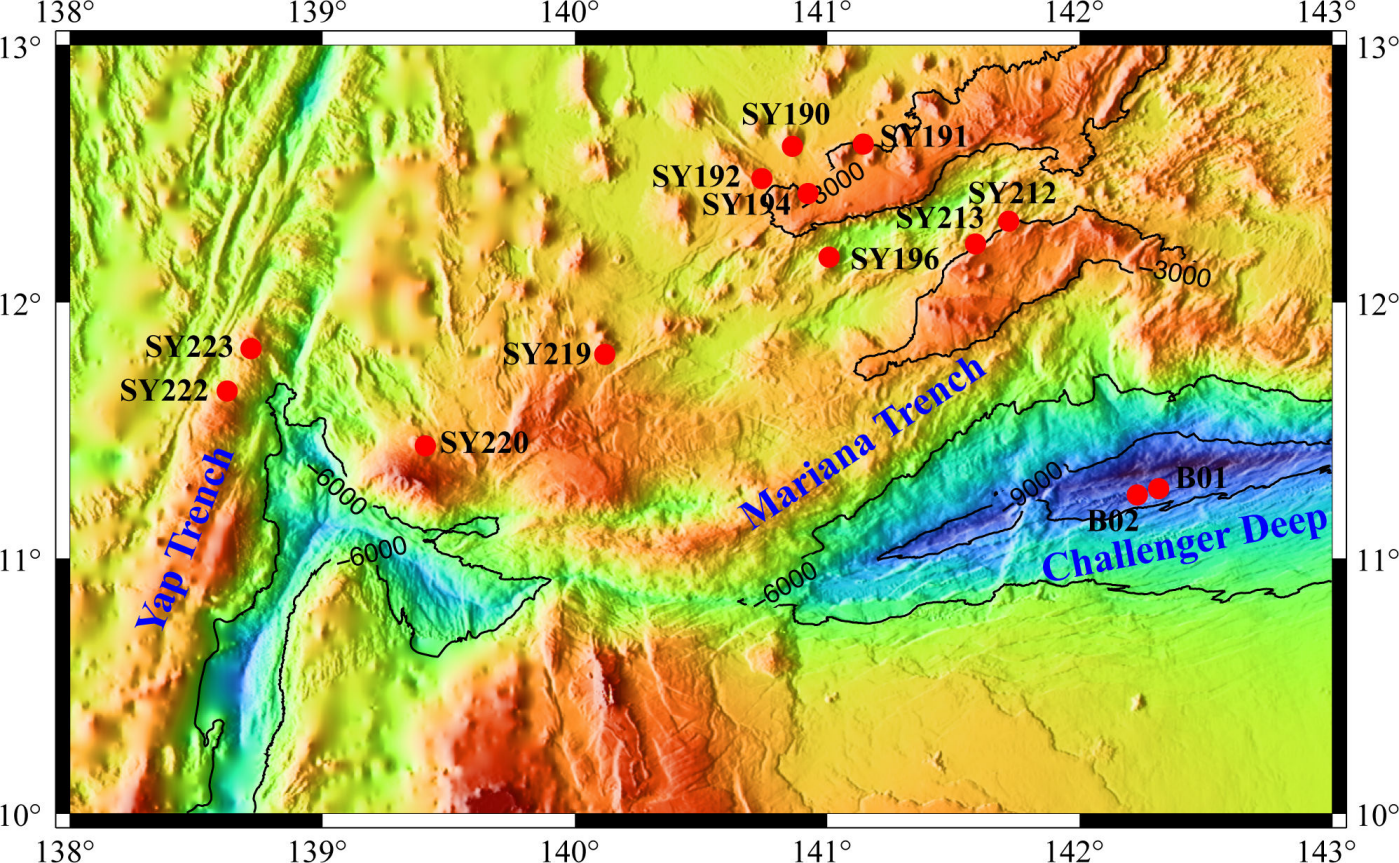
Map of the sampling stations along the island arc of the Yap and Mariana trenches.

Sediment samples were collected from the Yap Island Arc (YIA: SY222 and SY223), Yap-Mariana Junction area (YMJ: SY219 and SY220), Mariana Island Arc (MIA: SY190, SY191, SY192, SY194, SY212, and SY213), and the depression (the southwest Mariana rift: SY196; the Challenger Deep: B01 and B02) in the western Pacific Ocean. All the seamounts along the island arc were located about 2,670–3,456 m under water. Among them, Stn. SY220 in the YMJ was the highest one, approximately 3,258 m high and the summit at 282 m under water, while Stn. SY194 in the MIA was the lowest one with only 655 m high (Fig. 1). Generally, salinity was quite constant, and water temperature (1.54°C–2.18°C) decreased slightly with an increase of water depth (Table 1). Comparatively, significantly higher total nitrogen (TN) (0.32–1.00 mg/kg) (*P <* 0.05) and lower ammonia (NH_4_^+^) (0.77–1.11 mg/kg) (*P <* 0.05) contents were detected in the sediment of the depression than in the seamounts, except for Stns. SY212 and SY213; much higher total organic carbon (TOC) contents were shown in the seamounts than the depression. On a vertical scale, the highest concentrations of TOC (*P <* 0.05), NO_3_^−^ and NH_4_^+^ were present at the summit of the seamounts (Table 1).

**TABLE 1:** The environmental parameters and sequencing information of sediments collected from the island arc of the Yap and Mariana trenches^*a*^

Regions	Stns.	Lon. (°)	Lat. (°)	Temp. (°C)	Sal. (PSU)	Depth (m)	TN (mg/g)	TOC (mg/g)	NO_3_^-^ (mg/kg)	NH_4_^+^ (mg/kg)	Original reads	Quality reads	ASVs
Yap Island Arc (YIA)	SY222-flank	138.62	11.65	1.54	34.56	3,438	0.26	100.84	0.11	1.27	80,938	45,305	472
	SY223-summit	138.72	11.82	1.76	34.56	2,573	0.16	113.64	0.32	1.35	71,781	44,511	703
	flank			1.68	34.56	2,850	0.20	109.00	0.19	1.31	153,202	113,310	225
	base			1.64	34.57	3,206	0.26	102.23	0.02	1.33	5,547	3,425	243
Yap-Mariana junction (YMJ)	SY219-summit	140.12	11.80	1.60	34.56	3,135	0.20	104.56	0.04	1.34	138,688	92,089	334
	SY220-flank1	139.41	11.44	2.18	34.53	2,083	0.18	103.36	0.04	1.68	5,887	3,288	235
	Flank2			1.86	34.55	2,448	0.13	73.08	0.09	1.40	96,626	49,587	249
	base			1.77	34.56	2,670	0.17	66.58	0.10	1.45	88,275	53,240	653
Mariana Island Arc (MIA)	SY190-flank	140.87	12.61	–	–	2,758	0.21	102.68	0.59	1.29	149,509	28,866	199
	SY191-flank	141.14	12.62	–	–	3,247	0.56	72.34	0.42	1.24	16,603	8,930	248
	SY192-base	140.74	12.48	1.57	34.56	3,325	0.16	93.07	0.35	1.34	16,357	11,055	334
	SY194-base	140.92	12.42	1.75	34.53	2,974	0.19	100.47	0.19	1.27	6,408	3,808	250
	SY212-summit	141.72	12.32	1.69	34.56	2,800	0.12	15.84	0.28	1.63	85,369	52,807	587
	flank			1.62	34.57	3,143	0.14	5.58	0.14	1.45	165,220	39,020	269
	base			1.56	34.57	3,456	0.28	1.56	0.22	1.51	11,852	7,973	445
	SY213-summit	141.59	12.23	2.14	34.53	2,106	0.30	3.10	0.43	1.67	13,134	9,179	406
	flank			1.70	34.56	2,806	0.20	1.00	0.36	1.65	28,283	20,209	575
	base			1.53	34.57	3,423	0.50	3.37	0.01	1.13	7,995	4,691	294
Depression	SY196^*b*^	141.01	12.18	1.49	34.57	3,984	0.32	2.46	0.02	1.11	127,489	71,843	366
	B01^*c*^	142.31	11.27	–	–	9,946	0.78	4.44	0.31	0.77	72,021	21,574	177
	B02^*c*^	142.23	11.25	–	–	10,063	1.00	4.10	0.01	0.86	37,233	24,089	118

^*a*^ –, not detected.

^*b*^ From a rift.

^*c*^ From the Challenger Deep.

### Community composition and gene abundance

**Fig 2:**
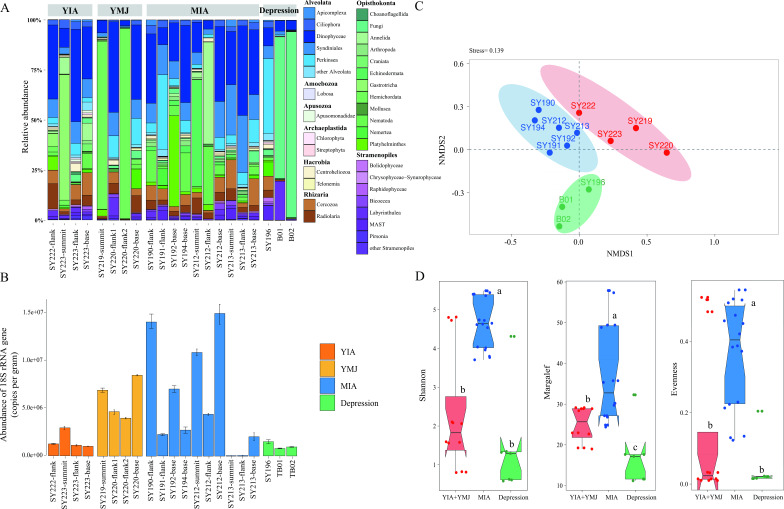
(**A**) Community structure of micro-eukaryotes at the super-group level; (**B**) abundance of micro-eukaryotic 18S rRNA gene with error bars representing standard deviation; (**C**) non-metric multidimensional scaling (NMDS) plot of micro-eukaryotes based on all ASVs; the data at different depths of the same station were integrated; (**D**) alpha diversity indices (Shannon, Margalef, and Evenness) of micro-eukaryotic communities in the MIA, YIA + YMJ, and the depression with box representing the lower quartile, median, and upper quartile. ab, ac, and bc, *P <* 0.05.

In total, 682,799 sequences and 3,612 amplicon sequence variants (ASVs) were generated with the maximum number found at Stn. SY223-summit (Table 1). The SAR (i.e., Stramenopiles, Alveolata, and Rhizaria) super-group and Matazoa dominated at all stations (Fig. 2A). Other super-groups (e.g., Amoebozoa, Apusozoa, and Hacrobia) together accounted for less than 10% in each sample. Dinophyceae, Syndiniales, and Perkinsea were dominant Alveolata assemblages, Radiolaria and Cercozoa were the main components of the Rhizaria group, and Labyrinthulea and MAST were the predominant Stramenopile group. Metazoa accounted for ~29.72% on average at all stations, comprised mainly of Arthropoda, Echinodermata, and Nematoda. Significantly lower proportion of Syndiniales and Perkinsea (*P <* 0.05) and higher proportion of Fungi (*P <* 0.05) were detected in the Challenger Deep than in the seamounts. On the vertical scale of the seamounts, lower proportions of Alveolata were generally present at the summit, while less Rhizavenn ria was present at the summit in the YIA and at the flank in the MIA.

Abundance of micro-eukaryotic 18S rRNA gene was significantly lower in the depression than in the seamounts (AMOSIM, *P <* 0.05) (Fig. 2B). Along the vertical profile, the highest gene abundance generally was detected at the base of the seamounts, except for Stn. 223. NMDS analysis showed that microbial community compositions from the depression were distinct from those of seamounts, and samples in the YIA and YMJ were clustered together, both of which were distinct from those in the MIA (Fig. 2C). The highest diversity indices (i.e., Shannon, Evenness, and Margalef) were found in the MIA (Fig. 2D) and were significantly different from those in the YIA + YMJ and the depression (AMOSIM, *P <* 0.05). In addition, more shared ASVs existed between seamounts than between the seamount and the depression. The highest and lowest specific ASVs appeared in the MIA and the depression, respectively (Fig. S1A). The indicative ASV leading to significant differences between the seamount and the depression was ASV3176 (Saccharomycetales, Fungi); however, it was mainly ASV 2994 (Harrimaniidae, Metazoa) that cause differences between seamounts in the YIA + YMJ and MIA (Fig. S1B).

### Vertical shifts of micro-eukaryotes along the seamounts

**Fig 3:**
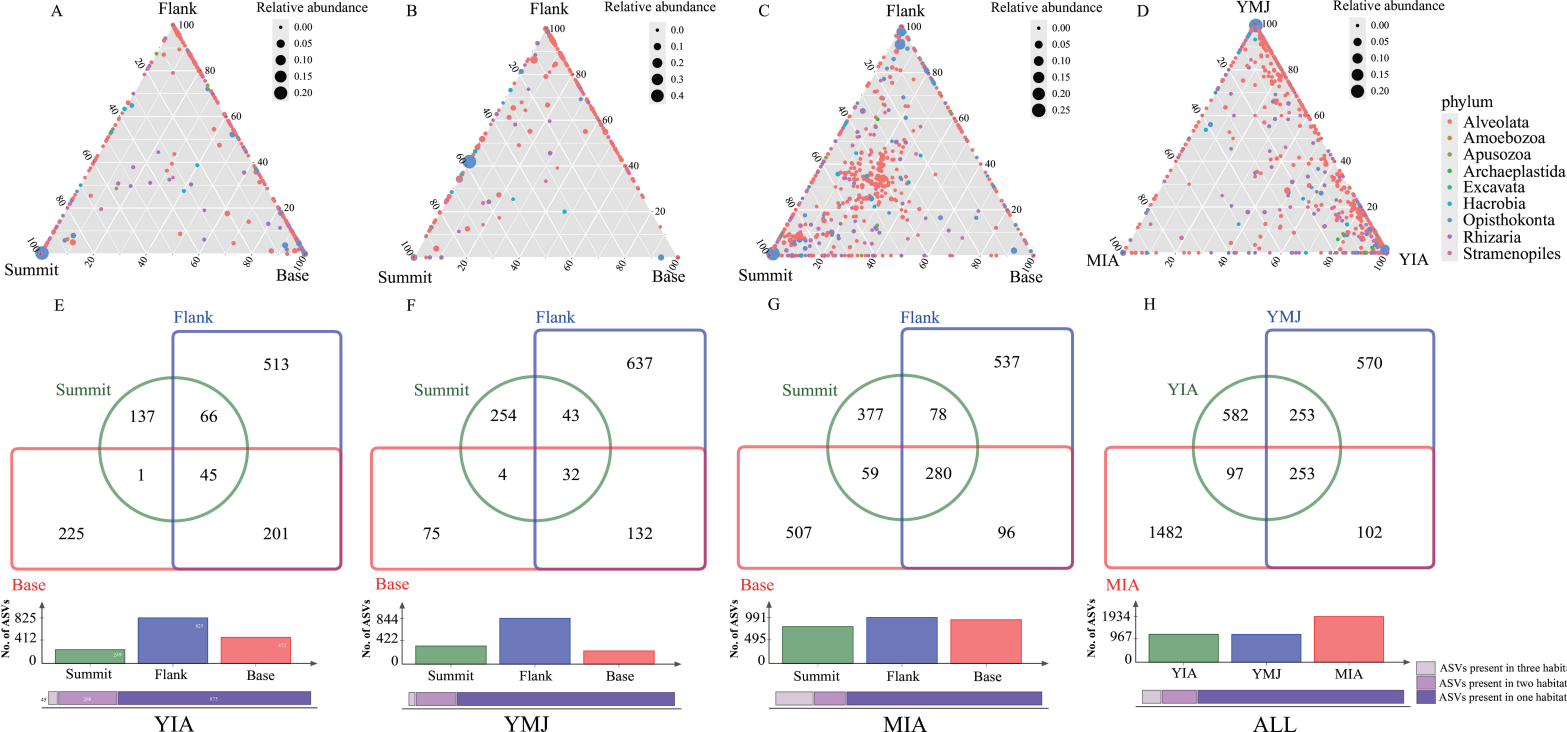
Ternary of microbial groups among the summit, flank, and base of the YIA (**A**), YMJ (**B**), and MIA (**C**) and the integrated seamount regions (**D**) (upper panel). Venn diagrams and bar charts based on the ASVs among the summit, flank, and base of the YIA (**E**), YMJ (**F**), and MIA (**G**) and the integrated seamount regions (**H**) (lower panel). Data from the summit, flank, and base of the seamount were integrated.

The distributions of micro-eukaryotic groups along the summit, flank, and base of seamounts in each region and integrated regions were illustrated by ternary and venn plots (Fig. 3). On the vertical scale, the distribution of ASVs was significantly different at the summit, flank, and base in the YIA (Fig. 3A) and the YMJ (Fig. 3B). For seamounts in the MIA, more ASVs with similar proportions were distributed near the center of the ternary plots (Fig. 3C). Among the seamounts in the three regions, most ASVs were enriched in the YIA and YMJ (Fig. 3D). Venn diagrams showed that the highest specific ASVs were always present at the flank of the seamounts in the YIA (Fig. 3E), YMJ (Fig. 3F), and MIA (Fig. 3G). More shared ASVs between the flank and base appeared in both YIA and YMJ, while those between the summit and flank in the MIA (Fig. 3E through G). Among seamounts in the three regions, YIA and YMJ had more shared ASVs (Fig. 3H).

### Driving forces of micro-eukaryotic communities

**Fig 4:**
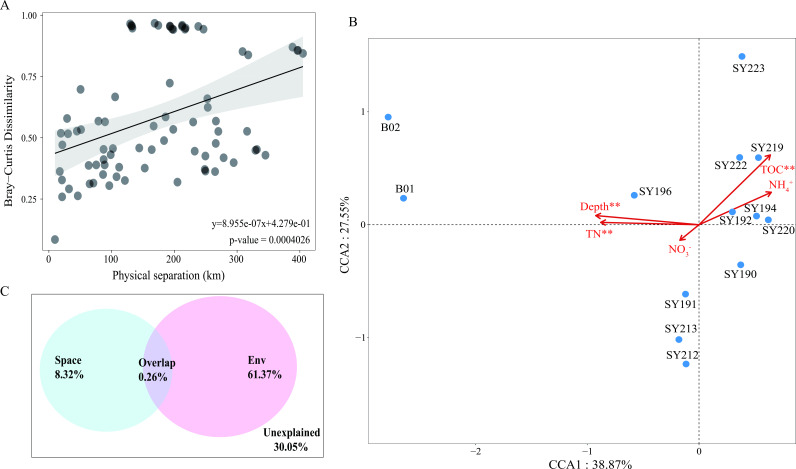
Driving forces of micro-eukaryotic community compositions based on all the ASVs. (**A**) Co-relations between community dissimilarities (Bray-Curtis distances) and geographic distances. (**B**) Canonical correspondence analysis of micro-eukaryotic communities with environmental variables. (**C**) Variation partitioning of micro-eukaryotic communities based on environmental variables and spatial factors. **P < 0.01.

Composition dissimilarity of the micro-eukaryotic communities increased with the geographical distance based on the distance-decay pattern (linear regression; slope = 8.955e^−7^; *P <* 0.01) (Fig. 4A). The neutral community model (NCM) analysis demonstrated the frequency of micro-eukaryotic ASVs fit rather weakly to the neutral model, and the majority of ASVs fell outside of the 95% CI of the neutral model prediction (Fig. S2), indicating that deterministic processes play a more critical role than stochasticity in the formation of micro-eukaryotic communities. After removing factors with VIF > 10, five environment parameters were used for canonical correspondence analysis (CCA). The first and second axes explained 38.87% and 27.55% of the total variance of the micro-eukaryotic community, respectively (Fig. 4B). Environmental variables, i.e., depth, TN, and TOC, significantly affected the community structure (499 permutation testing) (*P <* 0.01). VPA analysis showed that the total variation of micro-eukaryotic communities could be explained purely by spatial (8.32%) and environmental factors (61.37%) and explained simultaneously (0.26%) (Fig. 4C).

**Fig 5:**
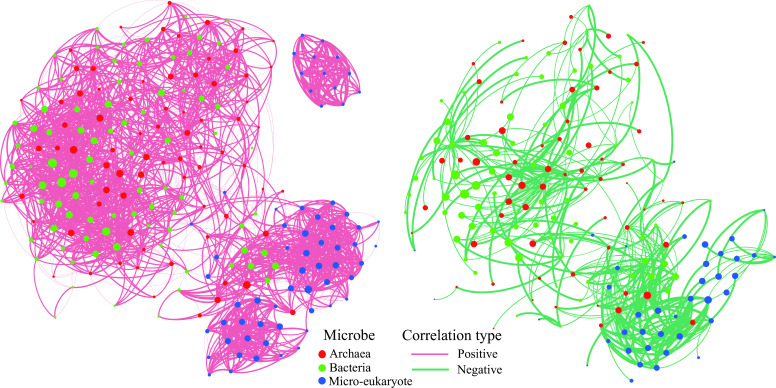
Co-occurrence networks of the combined micro-eukaryotic and prokaryotic ASVs. After filtering, any node that was no longer connected to another node was removed from the graph. Each network was filtered to present only positive edges (left) and only negative edges (right). Nodes are color coded to indicate archaeal, bacterial, and micro-eukaryotic groups and contain 300 members.

Inter-domain interactions of micro-eukaryotic and prokaryotic (unpublished data) ASVs were further performed to seek the driving force for the remaining unexplained variations (30.05%). Networks including positive and negative associations were presented by lines (edges) between individual ASVs (nodes). The network consisted of 300 nodes (100 archaeal ASVs, 100 bacterial ASVs, and 100 micro-eukaryotic ASVs) and 2,453 edges (Fig. 5). Most correlations for interactions between archaea/bacteria and micro-eukaryotes were positive, for example, between Ciliophora and Verrucomicrobia. However, negative correlations were also observed, e.g., between Ciliophora and Pseudomonadota and Actinomycetota.

### Potential trophic states of micro-eukaryotes

**Fig 6:**
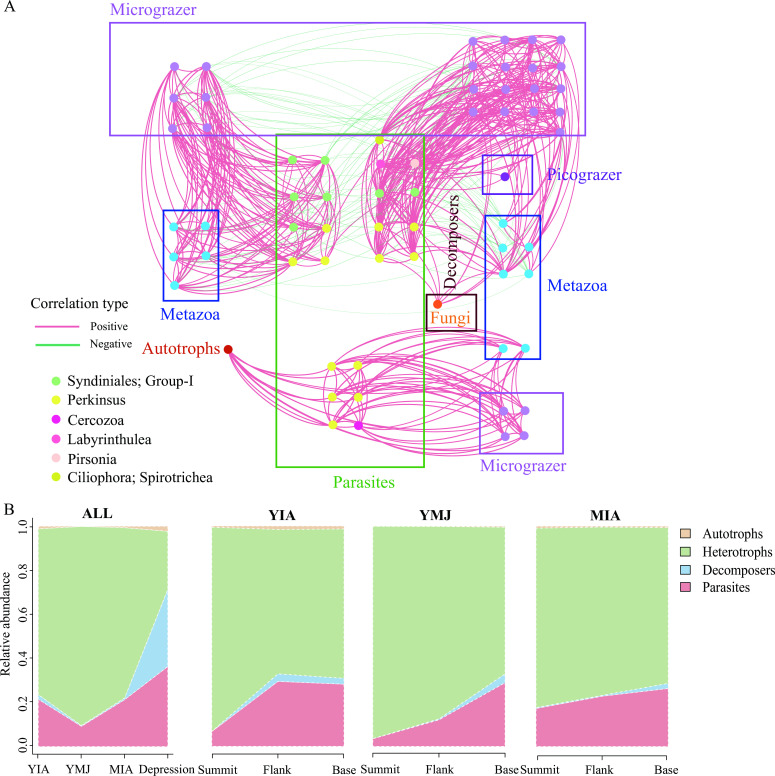
(**A**) Network diagram of highly significant connections (|*r*| > 0.6 and *P <* 0.05) among the top 100 ASVs (nodes). (**B**) The distribution of the trophic groups detected in the study. The trophic groups were based on the trophic status of ASVs as inferred from the literature (see Table S1).

The most abundant 100 micro-eukaryotic ASVs were identified as various micro/nano/picograzers, decomposers, and parasitic taxa (Table S1) and input for building networks to determine potential relationships among micro-eukaryotes with different trophic states (Fig. 6A). Heterotrophic (Dinophyceae and Metazoa) and parasitic (Syndiniales and Perkinsea) types were dominant in all the samples. Dinophyceae was a major group of micrograzer, including Radiolaria and Stramenopiles. Syndiniales showed co-occurrence with Alveolata (including Ciliophora and Dinophyceae) and a variety of Metazoa and Stramenopiles including MAST groups. Perkinsea co-occurred with Dinophyceae, Radiolaria, and Metazoa. Comparatively, heterotrophic types dominated in the seamounts and accounted for higher proportions at the summit; parasitic types were the second major group with the lowest proportions at the summit (Fig. 6B).

The distribution of different clades affiliated with parasitic Syndiniales and Perkinsea was further showed with the circos plot (Fig. S3). Dino-Group-I and II were highly represented with 5 and 18 clades identified, respectively. Clades 1, 2, and 4 accounted for higher proportions in the former group, while clades 10/11 and 15 were the major clades in the latter group. Dino-Group-III and IV were also present but with much less abundance. Significantly higher proportion of Perkinsea was found at Stn. SY196 (*P <* 0.05).

## DISCUSSION

### Distribution patterns and driving forces for micro-eukaryotes

Micro-eukaryotic communities from the seamounts were distinct from those in the depression, and those in the YIA and YMJ were closely clustered. These clear biogeographic distribution patterns were mainly shaped by deterministic processes as revealed from the significant distance-decay relationship. The importance of geographical distance has been reported from the open ocean (31), continental shelf (32), and coastal areas (33). Comparatively, environmental variability (61.37%) explained more of the community variation than the spatial variability (8.32%) based on the VPA. Notably, environmental factors, i.e., depth, TOC, and TN, had been reported as critical in other deep-sea ecosystems (34, 35). For the Magellan Seamount Chain in the western Pacific Ocean, clear depth effect on the vertical distribution of protist and fungal communities and the influence of TN content on the distribution of ciliate had been demonstrated (22, 36). We found fungi accounted for higher proportions in the depression and some assemblages of this group were decomposers with the capability of up-taking organic matter or be involved in nitrification and denitrification processes (37).

We further conducted co-occurrence network to solve the remaining unexplained variations (30.05%) in addition to the spatial and environmental effects. Both positive and negative interactions were exhibited between micro-eukaryotic and prokaryotic taxa, interpreted as cooperative/mutualistic and competitive/antagonistic relationships. The negative association between Radiolaria and Syndiniales/Actinomarinales, suggested a possible parasitic (e.g., Radiolaria and Syndiniales) or predatory (e.g., Radiolaria and Actinomarinales) interaction. Parasitism can alter the structure and dynamics of food webs, while predation is critical for releasing dissolved nutrients into the aquatic food web (38, 39). Positive interaction between Pseudomonadota and Ciliophora was very likely through vitamin exchange, N_2_-fixation, and photosymbiosis (24, 40). Diversified trophic states of microbes would be helpful for adaptation, diversification, and maintaining a steady balance of the ecosystem.

### Connectivity and trophic interaction of micro-eukaryotes

YMJ cuts across the Mariana Ridge and Yap Ridge; the close clustering of micro-eukaryotic communities in the YIA and YMJ and distinct from those of the MIA might be due to geographical proximity (41). More likely, there was a countercurrent flowing southward at a depth of 3,000–4,000 m on the eastern side of the YMJ (41), which may have facilitated the connectivity of microbial communities between the YIA and YMJ. On the other hand, the lower diversity, gene abundance, endemic species; and higher proportions of the parasitic types that appeared in the depression compared with the seamounts may be attributed to the low labile nutrient availability after longer burial time (42). Fungi more frequently appeared in the Challenger Deep, their predominance in the deepest zone of Mariana Trench might contribute to particle solubilization and remineralization of the marine snows (11). As for the Stn. SY196, it was located at the Southwest Mariana Rift, which is an active tectonic rift composed of the remnant arc West Mariana Ridge to the north with scarp heights up to 3,500 m (43), therefore, possibly sharing similar geological structure and nutrient concentration as the Challenger Deep, likely supporting similar micro-eukaryotic communities. However, this station is on a submarine rise of the rift at a depth of 3,984 m, so micro-eukaryotic community characteristics, e.g., diversity and the composition of metazoan groups, were similar to those in the seamounts. The reduced connectivity between seamounts and the depression and the enhanced community diversity in the seamounts reflected the seamount effect along the horizontal dimension, very likely due to the retention and advection effect of Taylor columns.

Seamounts are generally extinct underwater volcanoes (44), which has been suggested as oases for parasitic protists (26). The prevalence of parasitism in the seamounts might be caused by the wide spread of spores from parasitic Syndiniales and Perkinsida by diffusion and circulation (1). According to our network analysis, Syndiniale- and Perkinsida-related ASVs had a wide host range and variability, such as Metazoa (e.g., Annelida), dinoflagellates (e.g., Gyrodinium), MAST groups, and Radiolaria. Those host-promiscuous parasites, interacting with a large variety of other eukaryotes, could affect the food web (45). Many parasitic taxa were highly associated with analogous or matching functions, indicating that they possibly shared similar optimal niches (22). Parasitic fungi and coral parasitic cones were also found in the Magellan and Anton Dohrn seamounts previously (21, 46). In addition, heterotrophic Rhizaria showed a highly negative correlation with many other micro-eukaryotes, indicating the importance of predator-prey relationship in the communities. Rhizaria and Metazoa present in the current study likely played an important ecological role as parasite hosts and predators in the food web. Those diversified ecological relationships would be important in maintaining the ecological balance for micro-eukaryotic communities in this extreme biosphere. In addition, endemic saprophytic fungi, e.g., Blastocladiomycota and Entomophthoromycota, were found in the YIA and YMJ, and the latter was first spotted in the seamount. These indicative/endemic taxonomies might be a result of environmental selection and/or microbial adaptation to the different ecological niches.

### Seamount effect

Seamounts in the ocean could interrupt water flow resulting in changes of physical and chemical conditions, which are in turn expected to cause variations in microbial distribution, called “seamount effect.” The distinct distribution of micro-eukaryotes between the seamounts and the depression might be attributed to enclosed circulation cells created by the seamount (47) to reduce the community connectivity between seamount and the surrounding areas (48). Such horizontal seamount effect would cause microbial diversification, resulting in greater microbial diversity and species richness than the surrounding areas. Accordingly, higher diversity and more endemic species were detected in the seamounts than those in the depression, and low community connectivity between the two niches was revealed in the present study.

Additionally, the complex seamount topography would cause upwelling/downwelling around the seamount and may promote the movement of taxa between various water depths and thus enhance the vertical connectivity of micro-eukaryotic community and provide ecological opportunities to drive species adaption and diversification (22). It was not strange to find the lowest gene abundance present at the flank of the seamount, because the upwelling and downwelling flows along the seamount slope might cause persisting turbulence not a suitable niche for microbes (47), while the highest micro-eukaryotic gene abundance was generally present at the base of the seamount, consisting with the vertical pattern of protistan community (22). This was proposed to be attributed to the local potential hydrodynamic processes, such as the Antarctic intermediate water, the North Pacific deep water, and the lower circumpolar water, and the advection by those water flows might carry extra organic matter to foster the abundance and species richness of the benthos (49, 50). Much higher community connectivity occurred below the seamount summit in the YIA and YMJ, while occurring above the seamount base in the MIA. This vertical seamount effect very likely resulted from an uplift in the deep water as proved by the presence of abundant bottom-dwelling metazoan and upward transport of TOC in the Magellan Seamount Chain (36). The vertical connectivity generated by the seamount effect varied with different seamounts might be related to the upward flow caused by the current associated with eddies from different water layers encountering seamounts (51). The enhanced vertical connectivity could promote the taxon-taxon co-occurrence relationships and complexity (36), as demonstrated by the interaction of the network analysis. It should be noted that sediments used in our study may restrict the dispersal of species thus; the seamount effects might not be so obvious as for the pelagic communities. Nevertheless, these findings indicated a vertical connectivity of microbial communities around the deep seamount and expanded previous understanding of seamount effects on planktons from shallow seamounts (52) and intermediate-depth ridge (53).

Considering the complexity of topographic and hydrodynamic features of seamounts associated with diversified microbial communities, more environmental parameters especially the physical oceanographic conditions and the complex nonlinear dynamics should be considered to disentangle the driving force and mechanism shaping the microbial communities in this extreme biosphere in the future work.

### Conclusion

Our study clearly demonstrated the distinct biogeographic distribution patterns of micro-eukaryotes across a series of seamounts and illustrated the underlying community assembly mechanism. The horizontal seamount effect reduced community connectivity between the seamounts and the depression, while the upwelling/downwelling around the seamount enhanced vertical interactions of the micro-eukaryotic communities among the summit, flank, and base. In addition, the multiple trophic groups, e.g., grazers, decomposers, and parasitic taxa, added to the complexity of the micro-eukaryotic community and help maintain the ecological balance. In the future study, pelagic sampling over a larger geographical scale with detailed topographic and hydrodynamic information would help to elucidate the seamount effect on the geography and assembly of micro-eukaryotic communities in the deep-sea extreme biosphere.

## MATERIALS AND METHODS

### Sample collection and physicochemical parameter measurement

Sediment samples were collected from different seamounts located in the Yap Trench, Mariana Trench, and the depression of the western Pacific Ocean during cruise TS14 on R/V “Tan Suo Yi Hao” in September 2019 (Fig. 1). *In situ* hydrographical parameters (i.e., location, depth, temperature, and salinity) were recorded during sampling using the manned submersible, SHENHAI YONGSHI. The surface sediments (0–2 cm) were immediately stored at −80°C for further analysis. Sediment property analysis for nitrate (NO_3_^−^), ammonia, total organic carbon, and total nitrogen was conducted with approximately 5 g of frozen sediment at the Institute of Mountain Hazards and Environment, Chinese Academy of Sciences (Chengdu, Sichuan, China), according to Wang et al. (54). In brief, NO_3_^−^ and NH_4_^+^ were measured after 1 M HCl treatment followed by analysis with a colorimetric auto-analyzer (SEAL Analytical AutoAnalyzer 3, Germany). The TOC and TN concentrations were determined by over-drying the sediments at 105°C and then using an element analyzer (Elementar vario Macro cube, Germany). Each parameter was measured in triplicates, and values with standard error < 1% were treated as valid.

### DNA extraction, PCR amplification, and sequencing

Total DNAs were extracted from the surface sediment layers (0–2 cm) with the PowerSoil DNA Isolation Kit (MO BIO Laboratories Inc., Carlsbad, USA), according to the manufacturer’s protocol. The DNA was quantified with Qubit 2.0 (Life Technologies, USA), and the quality was checked via gel electrophoresis. DNA was then amplified using the FastStart High Fidelity PCR system (Roche) with the following universal primers: TAReuk454FWD1 (5ʹ-CCAGCA(G/C)C(C/T)GCGGTAATTCC-3ʹ) and REV3 (5′-ACTTTCGTTCTTGAT(C/T)(A/G)A-3′) (55) to target the V4 domain of the 18S rRNA gene. The PCR reaction was performed with an initial denaturation step of 95°C for 3 min, followed by 32 cycles of the following: 95°C for 30 s, 55°C for 30 s, and 72°C for 1 min, after which there was a final extension step of 72°C for 5 min. A negative control of double-distilled water was also performed during amplification in order to avoid reagent contamination. The paired-end sequencing of the amplicons was performed with an Illumina HiSeq PE250 sequencer (Novogene Co. Ltd., www.novogene.com).

### Quantitative PCR

The abundance of the 18S rRNA gene was quantified using the StepOnePlus quantitative PCR (qPCR) system (Applied Biosystems Inc., Carlsbad, CA, USA). Each qPCR reaction comprised of 10 µL 2× SYBR Premix Ex Taq II (TaKaRa Bio Inc., Shiga, Japan), 0.3 µM primer, 2 µL DNA as the template, 0.4 µL ROX reference dye, and water to a total of 20 µL. The qPCR reactions and calibrations were performed following a protocol described previously (25). In brief, as a positive control, a linear plasmid was constructed using the amplified PCR products and a TOPO-TA vector cloning kit (Invitrogen). Triplicate qPCR reactions were performed for each sample with efficiencies of 91.8%, and the gene copy number was normalized to the quantity of the gene.

### Bioinformatics analysis

After sequencing, primary processing of raw fastq files and demultiplexing of paired end sequences were performed using QIIME 2 (56). Trimming, primer sequence removal, sequence denoising, paired-end merging, chimeric sequence filtering, singleton removal, and sequence dereplication were completed with DADA2 (57). The representative sequences were picked and then compared with PR^2^ databases for micro-eukaryotes (58). Singletons and taxonomy assignment of amplicon sequence variants that were not affiliated with micro-eukaryotes were removed. The small-sized metazoan potentially adhering to the small particles of sediments were retained as part of the micro-eukaryotic communities. A filtered ASV table was generated for each sample with QIIME 2, and the diversity indices, i.e., Shannon, Evenness, and Margalef, were conducted with Paleontological Statistics (PAST) version 3 (59). The community structure of micro-eukaryotes was visualized via bar chart using the “ggplot2” packages in R version 3.5.3. The distributions of micro-eukaryotic groups among the summit, flank, and base of the seamounts were illustrated by ternary plots using “ggtern” package (60). The specific/endemic and shared ASVs were shown by venn diagram using “vegan” package (61). The similarity percentage analysis (SIMPER) test was performed to reveal the indicative ASVs responsible for the dissimilarity of community composition among different habitats.

ASVs were sorted into trophic groups by individually annotating them to a trophic status using the highest level of information. For ASVs affiliated to micro- and nanoplankton (e.g., dinoflagellates and ciliates), the confidence about their trophic role was high, as they had been also detected by microscopy. Conversely, ASVs affiliated with taxonomic groups impossible to detect with microscopy were annotated to higher taxonomic groups (e.g., family level) (62). Taxa belonging to groups Syndiniales and MAST (MArine STramenopiles) were considered symbionts and nanograzers, respectively (63, 64). The composition and distribution of trophic status among different habitats and at the summit, flank, and base of seamounts were visualized via stacking diagram using R version 3.5.3. Circos plots were generated using the “circlize” package (version 0.4.11) to show the distribution of parasitic Syndiniales and Perkinsea at different stations.

### Statistical analysis

The non-metric multidimensional scaling, based on the Bray-Curtis similarity index, was applied to analyze the similarity among different samples using PRIMER 5 (Plymouth Marine Laboratory, West Hoe, Plymouth, UK) (65). An analysis of similarities (ANOSIM) was conducted with Paleontological Statistics version 3 (59) to test whether there was a significant difference in the micro-eukaryotic community among the various sampling sites.

The distance-decay rate of the micro-eukaryotic community was calculated as the slope of linear least-squares regression for the relationship between geographic distance and micro-eukaryotic dissimilarity based on the Bray-Curtis metric using the “stats” package. To analyze the potential importance of stochastic processes to micro-eukaryotic community assembly, the Sloan neutral community model was employed to predict the relationships between ASV detection frequencies and their relative abundance across the metacommunity (66). The total fit to the neutral model was indicated by the parameter *R* ^2^. The NCM was developed as per R code in Chen et al. (67), which is available at https://github.com/Weidong-Chen-Microbial-Ecology/Stochastic-assembly-of-river-microeukaryotes.

Since the length of axis 1 of detrended correspondence analysis (DCA) > 3.0, canonical correspondence analysis was performed to analyze the associations between micro-eukaryotic communities and environmental factors. The relative impacting contributions of the spatial and environmental processes on micro-eukaryotes were tested through variance partitioning analysis in CANOCO v5.0 software (68) and illustrated with a venn diagram via the R version 3.5.3. Network analysis was conducted to explore the co-occurrence patterns within/between the taxa of micro-eukaryotes and prokaryotes (data not shown), within/between different trophic types of micro-eukaryotes as well. A similarity matrix was firstly generated by inputting a typical ASV matrix file, and then, the correlation matrix, r value, and *P* value were calculated using corr. test in the “psych” package of R version 3.5.3. ASVs which are strongly and significantly correlated (Spearman’s |*r*| > 0.6 and false discovery rate [FDR]-adjusted *P <* 0.05) were used to construct the networks using Gephi version 0.9.3 (69).

## Data Availability

All of the 18S rRNA gene sequences obtained from this study have been deposited in the National Center for Biotechnology Information (NCBI) Sequence Read Archive (SRA) under the accession number PRJNA917777.

## SUPPLEMENTAL MATERIAL

Supplemental file 1 spectrum.02069-23-s0001.docx Table S1, Fig. S1 to S3 legends, and supplemental references.

Supplemental file 2 spectrum.02069-23-s0002.tif Fig. S1.

Supplemental file 3 spectrum.02069-23-s0003.eps Fig. S2.

Supplemental file 4 spectrum.02069-23-s0004.tif Fig. S3.

ASM does not own the copyrights to Supplemental Material that may be linked to, or accessed through, an article. The authors have granted ASM a non-exclusive, world-wide license to publish the Supplemental Material files. Please contact the corresponding author directly for reuse.

## References

[1] de Vargas C , Audic S , Henry N , Decelle J , Mahé F , Logares R , Lara E , Berney C , Le Bescot N , Probert I , et al. . 2015. Eukaryotic plankton diversity in the sunlit ocean. Science 348:1261605. doi:10.1126/science.1261605 25999516

[2] Edgcomb VP . 2016. Marine protist associations and environmental impacts across trophic levels in the twilight zone and below. Curr Opin Microbiol 31:169–175. doi:10.1016/j.mib.2016.04.001 27092409

[3] Vellend M . 2010. Conceptual synthesis in community ecology. Q Rev Biol 85:183–206. doi:10.1086/652373 20565040

[4] Stegen JC , Lin X , Fredrickson JK , Chen X , Kennedy DW , Murray CJ , Rockhold ML , Konopka A . 2013. Quantifying community assembly processes and identifying features that impose them. ISME J 7:2069–2079. doi:10.1038/ismej.2013.93 23739053 PMC3806266

[5] Astorga A , Oksanen J , Luoto M , Soininen J , Virtanen R , Muotka T . 2012. Distance decay of similarity in freshwater communities: do macro- and microorganisms follow the same rules? Glob Ecol Biogeogr 21:365–375. doi:10.1111/j.1466-8238.2011.00681.x

[6] Finlay BJ . 2002. Global dispersal of free-living microbial eukaryote species. Science 296:1061–1063. doi:10.1126/science.1070710 12004115

[7] Azovsky A , Mazei Y . 2013. Do microbes have macroecology? Large-scale patterns in the diversity and distribution of marine benthic ciliates. Glob Ecol Biogeogr 22:163–172. doi:10.1111/j.1466-8238.2012.00776.x

[8] Azovsky AI , Tikhonenkov DV , Mazei YA . 2016. An estimation of the global diversity and distribution of the smallest eukaryotes: biogeography of marine benthic heterotrophic flagellates. Protist 167:411–424. doi:10.1016/j.protis.2016.07.001 27541705

[9] Foissner W . 2008. Protist diversity and distribution: some basic considerations. Biodivers Conserv 17:235–242. doi:10.1007/s10531-007-9248-5

[10] Pawlowski J , Christen R , Lecroq B , Bachar D , Shahbazkia HR , Amaral-Zettler L , Guillou L . 2011. Eukaryotic richness in the abyss: insights from pyrotag sequencing. PLoS One 6:e18169. doi:10.1371/journal.pone.0018169 21483744 PMC3070721

[11] Pernice MC , Giner CR , Logares R , Perera-Bel J , Acinas SG , Duarte CM , Gasol JM , Massana R . 2016. Large variability of bathypelagic microbial eukaryotic communities across the world’s oceans. ISME J 10:945–958. doi:10.1038/ismej.2015.170 26451501 PMC4796934

[12] Bik HM , Sung W , De Ley P , Baldwin JG , Sharma J , Rocha-Olivares A , Thomas WK . 2012. Metagenetic community analysis of microbial eukaryotes illuminates biogeographic patterns in deep-sea and shallow water sediments. Mol Ecol 21:1048–1059. doi:10.1111/j.1365-294X.2011.05297.x 21985648 PMC3261328

[13] Wessel P . 1997. Sizes and ages of seamounts using remote sensing: implications for intraplate volcanism. Science 277:802–805. doi:10.1126/science.277.5327.802

[14] Sonnekus MJ , Bornman TG , Campbell EE . 2017. Phytoplankton and nutrient dynamics of six south West Indian ocean seamounts. Deep Sea Res Part II Oceanogr 136:59–72. doi:10.1016/j.dsr2.2016.12.008

[15] Rogers AD . 2018. The biology of seamounts: 25 years on. Adv Mar Biol 79:137–224. doi:10.1016/bs.amb.2018.06.001 30012275

[16] Morato T , Hoyle SD , Allain V , Nicol SJ . 2010. Seamounts are hotspots of pelagic biodiversity in the open ocean. Proc Natl Acad Sci U S A 107:9707–9711. doi:10.1073/pnas.0910290107 20448197 PMC2906904

[17] Rowden AA , Schlacher TA , Williams A , Clark MR , Stewart R , Althaus F , Bowden DA , Consalvey M , Robinson W , Dowdney J . 2010. A test of the seamount oasis hypothesis: seamounts support higher epibenthic megafaunal biomass than adjacent slopes. Mar Ecol 31:95–106. doi:10.1111/j.1439-0485.2010.00369.x

[18] Genin A . 2004. Bio-physical coupling in the formation of zooplankton and fish aggregations over abrupt topographies. J Mar Syst 50:3–20. doi:10.1016/j.jmarsys.2003.10.008

[19] Clark MR , Rowden AA , Schlacher T , Williams A , Consalvey M , Stocks KI , Rogers AD , O’Hara TD , White M , Shank TM , Hall-Spencer JM . 2010. The ecology of seamounts: structure, function, and human impacts. Ann Rev Mar Sci 2:253–278. doi:10.1146/annurev-marine-120308-081109 21141665

[20] Zhang W , Liu J , Dong Y , Li X , Xu C , Xiao T , Pan H , Wu L-F . 2019. Archaeal community structure in sediments from a seamount in the Mariana volcanic arc. J Ocean Limnol 37:1197–1210. doi:10.1007/s00343-019-8044-x

[21] Yang S , Xu W , Gao Y , Chen X , Luo ZH . 2020. Fungal diversity in deep-sea sediments from Magellan seamounts environment of the western Pacific revealed by high-throughput Illumina sequencing. J Microbiol 58:841–852. doi:10.1007/s12275-020-0198-x 32876913

[22] Zhao R , Zhao F , Zheng S , Li X , Wang J , Xu K . 2022. Bacteria, protists, and fungi may hold clues of seamount impact on diversity and connectivity of deep-sea pelagic communities. Front Microbiol 13:773487. doi:10.3389/fmicb.2022.773487 35464911 PMC9024416

[23] Xu K . 2021. Exploring seamount ecosystems and biodiversity in the tropical western Pacific Ocean. J Ocean Limnol 39:1585–1590. doi:10.1007/s00343-021-1585-9

[24] Worden AZ , Follows MJ , Giovannoni SJ , Wilken S , Zimmerman AE , Keeling PJ . 2015. Environmental science. Rethinking the marine carbon cycle: factoring in the multifarious lifestyles of microbes. Science 347:1257594. doi:10.1126/science.1257594 25678667

[25] Zhang Y , Huang N , Wang M , Liu H , Jing H . 2021. Microbial eukaryotes associated with sediments in deep-sea cold seeps. Front Microbiol 12:782004. doi:10.3389/fmicb.2021.782004 35003010 PMC8740301

[26] Moreira D , López-García P . 2003. Are hydrothermal vents oases for parasitic protists? Trends Parasitol 19:556–558. doi:10.1016/j.pt.2003.09.013 14642764

[27] Torres-Beltrán M , Sehein T , Pachiadaki MG , Hallam SJ , Edgcomb V . 2018. Protistan parasites along oxygen gradients in a seasonally anoxic fjord: a network approach to assessing potential host-parasite interactions. Deep Sea Res Part II Oceanogr 156:97–110. doi:10.1016/j.dsr2.2017.12.026

[28] Lafferty KD , Allesina S , Arim M , Briggs CJ , De Leo G , Dobson AP , Dunne JA , Johnson PTJ , Kuris AM , Marcogliese DJ , Martinez ND , Memmott J , Marquet PA , McLaughlin JP , Mordecai EA , Pascual M , Poulin R , Thieltges DW . 2008. Parasites in food webs: the ultimate missing links. Ecol Lett 11:533–546. doi:10.1111/j.1461-0248.2008.01174.x 18462196 PMC2408649

[29] Crawford AJ , Beccaluva L , Serri G , Dostal J . 1986. Petrology, geochemistry and tectonic implications of volcanics dredged from the intersection of the Yap and Mariana trenches. Earth Planet Sci Lett 80:265–280. doi:10.1016/0012-821X(86)90110-X

[30] Davis RE , Moyer CL . 2008. Extreme spatial and temporal variability of hydrothermal microbial mat communities along the Mariana Island arc and southern Mariana back-arc system. J Geophys Res 113:B08S15. doi:10.1029/2007JB005413

[31] Zhao F , Filker S , Xu KD , Huang PP , Zheng S . 2020. Microeukaryote communities exhibit phyla-specific distance-decay patterns and an intimate link between seawater and sediment habitats in the western Pacific Ocean. Deep Sea Res Part I Oceanogr 160:103279. doi:10.1016/j.dsr.2020.103279

[32] Mars Brisbin M , Conover AE , Mitarai S . 2020. Influence of regional oceanography and hydrothermal activity on protist diversity and community structure in the Okinawa trough. Microb Ecol 80:746–761. doi:10.1007/s00248-020-01583-w 32948905

[33] Liu JW , Zhu SQ , Liu XY , Yao P , Ge TT , Zhang XH . 2020. Spatiotemporal dynamics of the archaeal community in coastal sediments: assembly process and co-occurrence relationship. ISME J 14:1463–1478. doi:10.1038/s41396-020-0621-7 32132664 PMC7242467

[34] Säwström C , Serrano O , Rozaimi M , Lavery PS . 2016. Utilization of carbon substrates by heterotrophic bacteria through vertical sediment profiles in coastal and estuarine seagrass meadows. Environ Microbiol Rep 8:582–589. doi:10.1111/1758-2229.12406 27188411

[35] Jing H , Zhang Y , Li Y , Zhu W , Liu H . 2018. Spatial variability of picoeukaryotic communities in the Mariana trench. Sci Rep 8:15357. doi:10.1038/s41598-018-33790-4 30337591 PMC6194128

[36] Zhao R , Zhao F , Feng L , Fang JK , Liu C , Xu K . 2023. A deep seamount effect enhanced the vertical connectivity of the planktonic community across 1000 m above summit. JGR Oceans 128. doi:10.1029/2022JC018898

[37] Ortiz-Álvarez R , Ortega-Arranz H , Ontiveros VJ , de Celis M , Ravarani C , Acedo A , Belda I . 2021. Emergent properties in microbiome networks reveal the anthropogenic disturbance of farming practices in vineyard soil fungal communities. Cold Spring Harbor Lab. doi:10.1128/mSystems.00344-21 PMC8269225 33947807

[38] Azam F , Fenchel T , Field JG , Gray JS , Meyer-Reil LA , Thingstad F . 1983. The ecological role of water-column microbes in the sea. Mar Ecol Prog Ser 10:257–263. doi:10.3354/meps010257

[39] Amundsen PA , Lafferty KD , Knudsen R , Primicerio R , Klemetsen A , Kuris AM . 2009. Food web topology and parasites in the pelagic zone of a subarctic lake. J Anim Ecol 78:563–572. doi:10.1111/j.1365-2656.2008.01518.x 19175443

[40] Caron DA , Countway PD , Jones AC , Kim DY , Schnetzer A . 2012. Marine protistan diversity. Ann Rev Mar Sci 4:467–493. doi:10.1146/annurev-marine-120709-142802 22457984

[41] Zhou C , Xu H , Xiao X , Zhao W , Yang J , Yang Q , Jiang H , Xie Q , Long T , Wang T , Huang X , Zhang Z , Guan S , Tian J . 2022. Intense abyssal flow through the Yap-Mariana junction in the western north Pacific. Geophys Res Lett 49. doi:10.1029/2021GL096530

[42] Fu L , Li D , Mi T , Zhao J , Liu C , Sun C , Zhen Y . 2020. Characteristics of the archaeal and bacterial communities in core sediments from southern Yap trench via in situ sampling by the manned submersible Jiaolong. Sci Total Environ 703:134884. doi:10.1016/j.scitotenv.2019.134884 31767325

[43] Sleeper JD , Martinez F , Fryer P , Stern RJ , Kelley KA , Ohara Y . 2021. Diffuse spreading, a newly recognized mode of crustal accretion in the southern Mariana trough backarc basin. Geosphere 17:1382–1404. doi:10.1130/GES02360.1

[44] Wessel P , Sandwell DT , Kim SS . 2010. The global seamount census. Oceanog 23:24–33. doi:10.5670/oceanog.2010.60

[45] Hudson PJ , Dobson AP , Lafferty KD . 2006. Is a healthy ecosystem one that is rich in parasites? Trends Ecol Evol 21:381–385. doi:10.1016/j.tree.2006.04.007 16713014

[46] Davies JS , Stewart HA , Narayanaswamy BE , Jacobs C , Spicer J , Golding N , Howell KL . 2015. Benthic assemblages of the Anton Dohrn seamount (NE Atlantic): defining deep-sea biotopes to support habitat mapping and management efforts with a focus on vulnerable marine ecosystems. PLoS One 10:e0124815. doi:10.1371/journal.pone.0124815 25992572 PMC4436255

[47] White M , Bashmachnikov I , Aristegui J , Martins A . 2007. Physical processes and seamount productivity, p 65–84. In Seamounts: ecology, fisheries and conservation. Blackwell Publishing, UK. doi:10.1002/9780470691953

[48] Giljan G , Kamennaya NA , Otto A , Becher D , Ellrott A , Meyer V , Murton BJ , Fuchs BM , Amann RI , Zubkov MV . 2020. Bacterioplankton reveal years-long retention of Atlantic deep-ocean water by the tropic seamount. Sci Rep 10:4715. doi:10.1038/s41598-020-61417-0 32170218 PMC7069937

[49] Lavelle JW , Mohn C . 2010. Motion, commotion, and biophysical connections at deep ocean seamounts. Oceanog 23:90–103. doi:10.5670/oceanog.2010.64

[50] Read J , Pollard R . 2017. An introduction to the physical oceanography of six seamounts in the southwest Indian Ocean. Deep Sea Res Part II Oceanogr 136:44–58. doi:10.1016/j.dsr2.2015.06.022

[51] Guo B , Wang W , Shu Y , He G , Zhang D , Deng X , Liang Q , Yang Y , Xie Q , Wang H , Wang J . 2020. Observed deep anticyclonic cap over Caiwei Guyot. JGR Oceans 125:e2020JC016254. doi:10.1029/2020JC016254

[52] Eriksen CC . 1998. Internal wave reflection and mixing at Fieberling Guyot. J Geophys Res 103:2977–2994. doi:10.1029/97JC03205

[53] Meredith MP , Meijers AS , Naveira Garabato AC , Brown PJ , Venables HJ , Abrahamsen EP , Jullion L , Messias M . 2015. Circulation, retention, and mixing of waters within the Weddell-Scotia confluence, southern Ocean: the role of stratified Taylor columns. JGR Oceans 120:547–562. doi:10.1002/2014JC010462

[54] Wang JP , Wu YH , Zhou J , Bing HJ , Sun HY . 2016. Carbon demand drives microbial mineralization of organic phosphorus during the early stage of soil development. Biol Fertil Soils 52:825–839. doi:10.1007/s00374-016-1123-7

[55] Stoeck T , Bass D , Nebel M , Christen R , Jones MDM , Breiner H-W , Richards TA . 2010. Multiple marker parallel tag environmental DNA sequencing reveals a highly complex eukaryotic community in marine anoxic water. Mol Ecol 19 Suppl 1:21–31. doi:10.1111/j.1365-294X.2009.04480.x 20331767

[56] Caporaso JG , Kuczynski J , Stombaugh J , Bittinger K , Bushman FD , Costello EK , Fierer N , Peña AG , Goodrich JK , Gordon JI , et al. . 2010. QIIME allows analysis of high-throughput community sequencing data. Nat Methods 7:335–336. doi:10.1038/nmeth.f.303 20383131 PMC3156573

[57] Callahan BJ , McMurdie PJ , Rosen MJ , Han AW , Johnson AJA , Holmes SP . 2016. DADA2: high-resolution sample inference from Illumina amplicon data. Nat Methods 13:581–583. doi:10.1038/nmeth.3869 27214047 PMC4927377

[58] Guillou L , Bachar D , Audic S , Bass D , Berney C , Bittner L , Boutte C , Burgaud G , de Vargas C , Decelle J , et al. . 2013. The protist ribosomal reference database (PR2): a catalog of unicellular eukaryote small sub-unit rRNA sequences with curated taxonomy. Nucleic Acids Res 41:D597–604. doi:10.1093/nar/gks1160 23193267 PMC3531120

[59] Hammer Y , Harper DA , Ryan PD . 2001. Past: paleontological statistics software package for education and data analysis. Palaeontol Electron 4:1–9. https://paleo.carleton.ca/2001_1/past/past.pdf.

[60] Hamilton NE , Ferry M . 2018. ggtern: ternary diagrams using ggplot2. J Stat Soft 87:1–17. doi:10.18637/jss.v087.c03

[61] Oksanen J , Blanchet FG , Kindt R , Legendre P , Minchin PR , O’hara RB , Simpson GL , Solymos P , Stevens MHH , Wagner H . 2010. Community ecology package, version, 2. Package ‘vegan.’ https://cran.ism.ac.jp/web/packages/vegan/vegan.pdf.

[62] Bachy C , Dolan JR , López-García P , Deschamps P , Moreira D . 2013. Accuracy of protest diversity assessments: morphology compared with cloning and direct pyrosequencing of the 18S rRNA genes and ITS regions using the conspicuous tintinnid ciliates as a case study. ISME J 7:244–255. doi:10.1038/ismej.2012.106 23038176 PMC3554406

[63] Massana R , Terrado R , Forn I , Lovejoy C , Pedrós-Alió C . 2006. Distribution and abundance of uncultured heterotrophic flagellates in the world oceans. Environ Microbiol 8:1515–1522. doi:10.1111/j.1462-2920.2006.01042.x 16913912

[64] Skovgaard A . 2014. Dirty tricks in the plankton: diversity and role of marine parasitic protists. Acta Protozool 53:51–62. doi:10.4467/16890027AP.14.006.1443

[65] Clarke KR , Warwick RM. 2001. Changes in marine communities: an approach to statistical analysis and interpretation. Primer-E Ltd. Plymouth, UK. https://plymsea.ac.uk/id/eprint/7656.

[66] Sloan WT , Lunn M , Woodcock S , Head IM , Nee S , Curtis TP . 2006. Quantifying the roles of immigration and chance in shaping prokaryote community structure. Environ Microbiol 8:732–740. doi:10.1111/j.1462-2920.2005.00956.x 16584484

[67] Chen W , Ren K , Isabwe A , Chen H , Liu M , Yang J . 2019. Stochastic processes shape microeukaryotic community assembly in a subtropical river across wet and dry seasons. Microbiome 7:148. doi:10.1186/s40168-019-0763-x 31727140 PMC6857158

[68] Šmilauer P , Lepš J . 2014. Multivariate analysis of ecological data using CANOCO 5. Cambridge University Press, New York, NY, USA.

[69] Bastian M , Heymann S , Jacomy M . 2009. Gephi: an open source software for exploring and manipulating networks. ICWSM 3:361–362. doi:10.1609/icwsm.v3i1.13937

