# Supplementary material for: Geographical distribution and driving force of micro-eukaryotes in the seamount sediments along the island arc of the Yap and Mariana trenches
Source: Microbiol Spectr. 2023 Nov 9;11(6):e02069-23. doi: 10.1128/spectrum.02069-23 (PMC10714776; doi:10.1128/spectrum.02069-23)
Supplement: Supplemental file 1 — Table S1, Fig. S1 to S3 legends, and supplemental references. [file spectrum.02069-23-s0001.docx]

**Table S1** Annotated taxa, major trophic roles and abundance of the top ASVs in the sediments along the island arc of the Yap and Mariana trenches.

| Super groups | Groups | Number of ASVs | Major trophic role | References |
| --- | --- | --- | --- | --- |
| Alveolata | Apicomplexa | 48 | Parasites | Skovgaard, 2014 (1) |
|  | Ciliophora | 196 | Micrograzers (Heterotrophs, Mixotrophs) & parasites | Lynn, 2008 (2) |
|  | Dinophyceae | 1054 | Micrograzers (Heterotrophs, Mixotrophs) & parasites | Sherr and Sherr, 2007 (3) |
|  | Syndiniales | 425 | Parasites | Guillou et al., 2008 (4) |
|  | Perkinsea | 84 | Parasites | Park et al., 2004 (5) |
| Amoebozoa | Lobosa | 17 | Micrograzers & some parasites | Lesen et al., 2010 (6) |
| Apusozoa | Apusomonadidae | 34 | Picograzers (nanoﬂagellates) | Lee, 2006 (7) |
| Archaeplastida | Chlorophyta | 41 | Autotrophs | Not et al., 2012 (8) |
|  | Streptophyta | 22 | Autotrophs | Not et al., 2012 (8) |
| Hacrobia | Centroheliozoa | 47 | Pico/nanograzers | Burki et al., 2009 (9) |
|  | Telonemia | 11 | Picograzers | Klaveness et al., 2005 (10) |
| Opisthokonta | Choanoflagellida | 17 | Picograzers (nanoﬂagellates) | King et al., 2009 (11) |
|  | Fungi | 99 | Decomposers & parasites | Manohar and Raghukumar, 2013 (12) |
|  | Metazoa | 384 | Heterotrophs | King et al., 2009 (11) |
| Rhizaria | Cercozoa | 330 | Parasites | Chantangsi and Leander, 2010 (13) |
|  | Radiolaria | 332 | Micrograzers | Kishi et al., 2008 (14) |
| Stramenopiles | Bolidophyceae | 2 | Autotrophs | Brown and Sorhannus, 2010 (15) |
|  | Chrysophyceae-Synurophyceae | 26 | Autotrophs & mixotrophs | Brown and Sorhannus, 2010 (15) |
|  | Raphidophyceae | 13 | Picograzers | Clough et al., 2006 (16) |
|  | Bicoecea | 31 | Picograzers | Filker et al., 2017 (17) |
|  | Labyrinthulea | 85 | Parasites | Raghukumar, 2002 (18) |
|  | MAST | 70 | Picograzers (nanoﬂagellates) | Massana et al., 2006 (19) |
|  | Pirsonia | 38 | Parasites | Skovgaard, 2014 (1) |

**Figure S1.** (A) Venn diagrams based on micro-eukaryotic taxa between different regions. (B) The “indicative ASVs” between two regions were calculated based on the SIMPER analysis with ASVs that contributed to ≥1% of dissimilarities. For each ASV, its name and the lowest recognized classification was labeled.

**Figure S2.** Fit of the Sloan neutral community model (NCM) to micro-eukaryotic ASV data on the horizontal (upper panel) and vertical (lower panel) scales. The solid blue lines indicate the best fit to the NCM, and the dashed blue lines represent 95% confidence intervals around the model predictions. ASVs that occur more and less frequently than predicted by the NCM were shown in different colors. Rsqr (R^2^) indicated the fit of the model, Nm indicated the metacommunity size times immigration.

**Figure S3.** Circos plot showing the distribution of the dominant clades of parasitic Syndiniales and Perkinsea at all the stations along the island arc of the Yap and Mariana trenches.

**References**

1. Skovgaard A. 2014. Dirty tricks in the plankton: diversity and role of marine parasitic protists. Acta Protozool 53: 51-62. https://doi.org/10.4467/16890027AP.14.006.1443.
2. Lynn D. 2008. The ciliated protozoa: characterization, classification and guide to the literature, 3rd Edn. New York, NY: Springer. https://doi.org/10.1007/978-1-4020-8239-9_17.
3. Sherr EB, Sherr BF. 2007. Heterotrophic dinoflagellates: a significant component of microzooplankton biomass and major grazers of diatoms in the sea. Mar Ecol Prog Ser 352: 187-197. https://doi.org/10.3354/meps07161.
4. Guillou L, Viprey M, Chambouvet A, Welsh RM, Kirkham AR, Massana R, Scanlan DJ, Worden AZ. 2008. Widespread occurrence and genetic diversity of marine parasitoids belonging to Syndiniales (Alveolata). Environ Microbiol 10: 3349-3365. https://doi.org/10.1111/j.1462-2920.2008.01731.x
5. Park MG, Yih W, Coats DW. 2004. Parasites and phytoplankton, with special emphasis on dinoflagellate infections. J Eukaryot Microbiol 51: 145-155. https://doi.org/10.1111/j.1550-7408.2004.tb00539.x.

6. Lesen AE, Juhl AR, Anderson OR. 2010. Heterotrophic microplankton in the lower Hudson River Estuary: potential importance of naked, planktonic amebas for bacterivory and carbon flux. Aquat Microb Ecol 61: 45-56. https://doi.org/10.3354/ame01434.

7. Lee W. 2006. Some free-living heterotrophic flagellates from marine sediments of tropical Australia. Ocean Sci. J. 41, 75-95. 10.1007/BF03022413.

8.Not F, Siano R, Kooistra WHCF, Simon N, Vaulot D, Probert I. 2012. Diversity and ecology of eukaryotic marine phytoplankton. Adv Bot Res 64: 1-53. https://doi.org/10.1016/B978-0-12-391499-6.00001-3.

9. Burki F, Inagaki Y, Bråte J, Archibald JM, Keeling PJ, Cavalier-Smith T, Sakaguchi M, Hashimoto T, Horak A, Kumar S, Klaveness D. Jakobsen KS, Pawlowski J, Shalchian-Tabrizi K. 2009. Large-scale phylogenomic analyses reveal that two enigmatic protist lineages, telonemia and centroheliozoa, are related to photosynthetic chromalveolates. Genome Bio Evol 1: 231-238. https://doi.org/10.1093/gbe/evp022.

10. Klaveness D, Shalchian-Tabrizi K, Thomsen HA, Eikrem W, Jakobsen KS. 2005. *Telonema antarcticum* sp. nov., a common marine phagotrophic flagellate. Int J Syst Evol Microbiol.55: 2595-2604. https://doi.org/10.1099/ijs.0.63652-0.

11. King N, Young SL, Abedin M, Carr M, Leadbeater BS. 2009. The choanoflagellates: heterotrophic nanoflagellates and sister group of the metazoa. Cold Spring Harb Protoc pdb-emo116. https://doi.org/10.1101/pdb.emo116.

12. Manohar CS, Raghukumar C. 2013. Fungal diversity from various marine habitats deduced through culture-independent studies. FEMS Microbiol Lett 341: 69-78. https://doi.org/10.1111/1574-6968.12087.

13. Chantangsi C, Leander BS. 2010. An SSU rDNA barcoding approach to the diversity of marine interstitial cercozoans, including descriptions of four novel genera and nine novel species. Int J Syst Evol Micr 60: 1962-1977. https://doi.org/10.1099/ijs.0.013888-0.

14. Brown JW, Sorhannus U. 2010. A molecular genetic timescale for the diversification of autotrophic stramenopiles (Ochrophyta): substantive underestimation of putative fossil ages. PLoS ONE 5: e12759. https://doi.org/10.1371/journal.pone.0012759.

15. Kishi Y, Watanabe Y, Ishida H, Yamamoto Y, Nakata K. 2008. Ecosystem model of a deep-sea plankton community for CO_2_ ocean sequestration. OCEANS 2008-MTS/IEEE Kobe Techno-Ocean, 1-6. https://doi.org/10.1109/OCEANSKOBE.2008.4530998.

16. Clough J, Strom S. 2005. Effects of Heterosigma akashiwo (Raphidophyceae) on protist grazers: laboratory experiments with ciliates and heterotrophic dinoflagellates. Aquat Microb Ecol 39: 121-134. https://doi.org/10.3354/ame039121.

17.Filker S, Forster D, Weinisch L, Mora-Ruiz M, González B, Farías ME, Rosselló-Móra R, Stoeck T. 2017. Transition boundaries for protistan species turnover in hypersaline waters of different biogeographic regions. Environ Microbiol 19: 3186-3200. https://doi.org/10.1111/1462-2920.13805.

18. Raghukumar S. 2002. Ecology of the marine protists, the Labyrinthulomycetes (Thraustochytrids and Labyrinthulids). Eur J.Protistol 38: 127-145. https://doi.org/10.1078/0932-4739-00832.

19. Massana R, Terrado R, Forn I, Lovejoy C, Pedrós-Alió C. 2006. Distribution and abundance of uncultured heterotrophic flagellates in the world oceans. Environ Microbiol 8: 1515-1522. https://doi.org/10.1111/j.1462-2920.2006.01042.x.
