# Supplementary figures and images for: Geographical distribution and driving force of micro-eukaryotes in the seamount sediments along the island arc of the Yap and Mariana trenches
Source: Microbiol Spectr. 2023 Nov 9;11(6):e02069-23. doi: 10.1128/spectrum.02069-23 (PMC10714776; doi:10.1128/spectrum.02069-23)

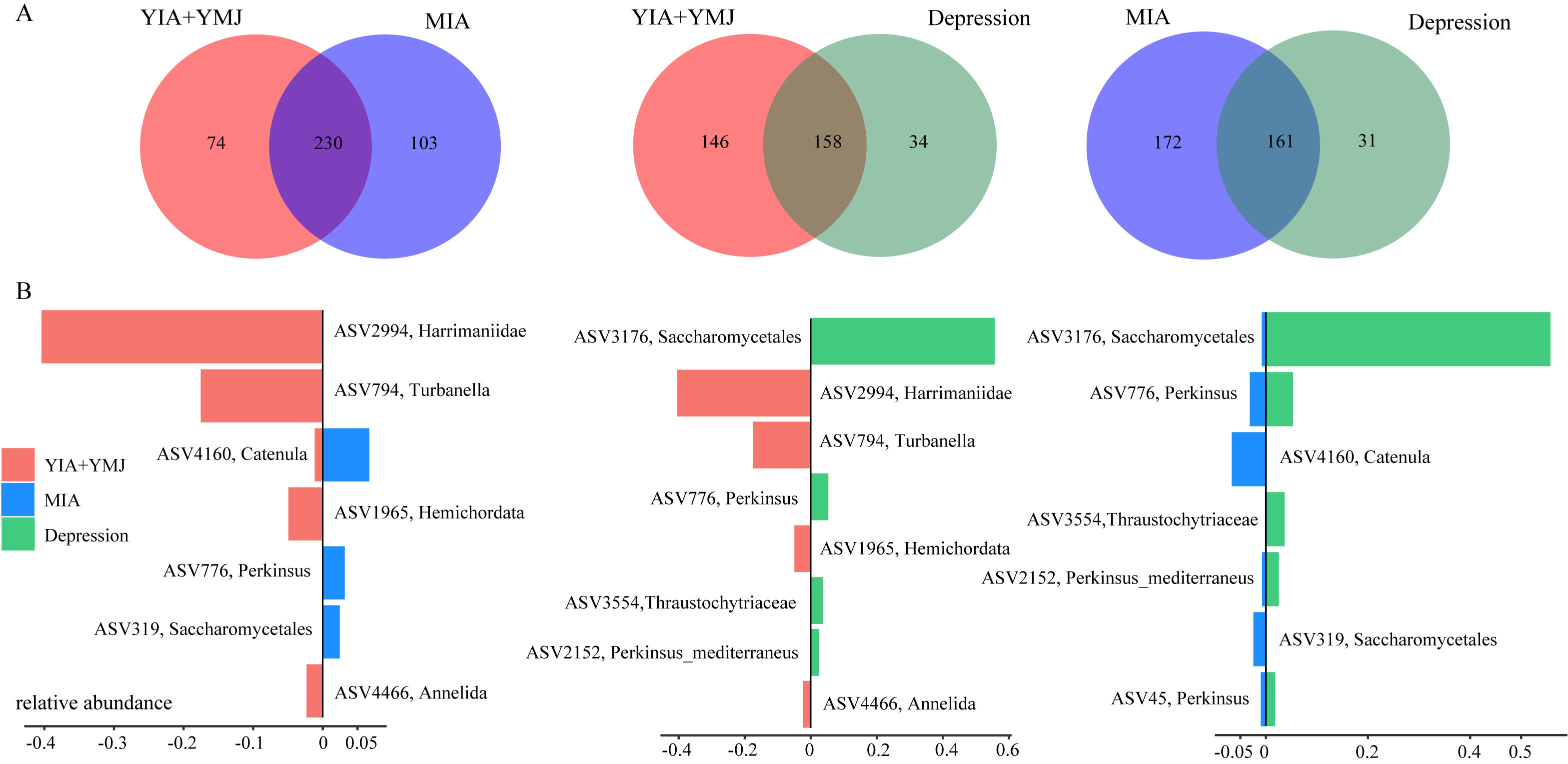

Supplement: Supplemental file 2 — Fig. S1. [file spectrum.02069-23-s0002.tif]
